# Psychometric properties and diagnostic accuracy of the short form of the geriatric anxiety scale (GAS-10)

**DOI:** 10.1186/s12877-021-02350-3

**Published:** 2021-06-30

**Authors:** Leonardo Carlucci, Matteo Balestrieri, Elisa Maso, Alessia Marini, Nadia Conte, Michela Balsamo

**Affiliations:** 1 Department of Psychological Sciences, Health and Territory, “G. d’Annunzio” University of Chieti-Pescara, via dei Vestini, 31 66100 Chieti, Italy; 2 Unit of Psychiatry, Department of Medicine (DAME), University of Udine, Udine, Italy; 3 Psychiatric Clinic, Healthcare and University Integrated Trust, Udine, Italy

**Keywords:** Anxiety, IRT, Geriatric, GAD, Elderly

## Abstract

**Background:**

Anxious symptoms have a negative impact on different aspects of the elderly’s quality of life, ranging from the adoption of unhealthy lifestyle behaviours to an increased functional impairment and a greater physical disability. Different brief assessment instruments have been developed as efficacy measures of geriatric anxiety in order to overcome psychometric weaknesses of its long form. Among these, the 10-item Geriatric Anxiety Scale (GAS-10) showed strong psychometric properties in community-dwelling samples. However, its diagnostic accuracy is still unexplored, as well as its discriminative power in clinical samples.

**Methods:**

In the present study, we explored the psychometric performance of the GAS-10 in the elderly through Item Response Theory in a sample of 1200 Italian community-dwelling middle-aged and elderly adults (53.8% males, mean age = 65.21 ± 9.19 years). Concurrent validity, as well as diagnostic accuracy, was examined in a non-clinical sample (*N* = 229; 46.72% males) and clinical sample composed of 35 elderly outpatients (74.28% females) with Generalized Anxiety Disorder (GAD).

**Results:**

The GAS-10 displayed good internal construct validity, with unidimensional structure and no local dependency, good accuracy, and no signs of Differential Item Functioning (DIF) or measurement bias due to gender, but negligible due to the age. Differences in concurrent validity and diagnostic accuracy among the long form version of the GAS and the GAS-10 were not found significant. The GAS-10 may be more useful than the longer versions in many clinical and research applications, when time constraints or fatigue are issues.

**Conclusion:**

Using the ROC curve, the GAS-10 showed good discriminant validity in categorizing outpatients with GAD disorder, and high anxiety symptoms as measured by the GAS-SF cut-off. The stable cut-off point provided could enhance the clinical usefulness of the GAS-10, which seems to be a promising valid and reliable tool for maximize diagnostic accuracy of geriatric anxiety symptoms.

## Introduction

In Europe, anxiety disorders are one of the most common mental health problems among elderly, with lifetime prevalence estimates ranging from 20.1% in Italy to 32.6% in England [1, 2]. This prevalence is probably underestimated, considering that subsyndromal manifestations of anxious symptoms probably range from 15 to 52.3% in community-living older adults [3, 4]. With an increasingly ageing population, the socio-economic costs of mental health problems – such as anxiety disorders – are growing significantly [3], especially in countries like Italy with low fertility rates and a high life expectancy. As of January 1st 2020, 13.9 million Italians (23.1% of the population) were over 65 years of age, and this percentage continues to increase annually [5]. By 2050 this percentage is expected to reach nearly 30% in most of the western countries [6].

Despite its impact and the high prevalence among the elderly population, anxiety remains often undiagnosed [7, 8]. A possible explanation lies on the specific characteristics of anxious symptoms in later life. Indeed, older adults typically report an increased sensitivity to several types of somatic stimuli and more physical complaints than younger adults, which could be mistaken for psychosomatic symptoms [9]. Moreover, anxious symptoms are often comorbid with other medical conditions and depressive disorders, worsening their prognosis and making the differential diagnosis difficult [10, 11]. Anxiety has also a substantial socio-economic burden, due to an increased use of health services among those who report greater symptoms [12]. Thus, both clinicians and mental health practitioners need valid, reliable measures of anxious symptoms specifically tailored for this population and carefully developed to consider the clinical manifestations of late-life anxiety.

Self-report measures are commonly used to assess anxious symptoms in elderly thanks to their easy administration and to the reduced respondent burden compared to other assessment methods [13]. In their recent work, Balsamo and colleagues [13] reviewed 12 self-report instruments that are commonly used to assess anxiety in later life, some of which are specifically tailored for older population, while others have been validated on geriatric population only later. The first group includes the Geriatric Anxiety Scale (GAS) [14], the Adult Manifest Anxiety Scale (AMAS), [15] the Geriatric Anxiety Inventory (GAI), [16] and the Worry Scale (WS), [17]; while among the second group there are the State-Trait Anxiety Inventory (STAI), [18] the Beck Anxiety Inventory (BAI), [19] the Penn State Worry Questionnaire (PSWQ), [20], and the State-Trait Inventory for Cognitive and Somatic Anxiety (STICSA), [21]. They varied depending on the design format (Likert scales vs. dichotomous answers), the length (from 16 to 88 items), and the underlying factor structures (unidimensional vs. multidimensional). However, despite their popularity in contexts whereby assessing anxiety in older adults is required, some of these measures lack good psychometric properties, especially those not originally intended for older adults [13].

Among those instruments specifically tailored for older adults, the Geriatric Anxiety Scale (GAS) is among the most promising ones. Characterized by good to excellent psychometric properties, GAS was developed by Segal and colleagues in 2010. It is composed of 30 items (even though 5 items are intended for clinical purposes and therefore they are not included in the final score) rated on a 4-point Likert scale, ranging from 0 (not at all) to 3 (all of the time). The GAS investigates three dimensions of anxiety, namely Somatic, Cognitive, and Affective ones, and this factorial structure has been confirmed in recent research [22]. The original version of the instrument has been validated on a large sample of community dwelling older adults [14] and on a sample of medically ill older adults [23]. To date, the GAS is one of the most widely-adopted instrument for the measurement of anxiety in geriatric population, as evidenced by its translation in many languages: Arabic [24] Chinese [25], German [26], Italian [27], Persian [28], and Turkish [29]. Furthermore, psychometric properties of the GAS are good: it demonstrated excellent internal consistency (α = 0.88–0.93) in non-clinical population and significant convergent and discriminant validity [13].

Due to the specificity of the target population, there is a need for development and validation of short-forms of geriatric anxiety questionnaires, that allow researchers and clinicians to collect necessary symptom data with less respondent burden [30]. Shorter versions may also help in reducing missing data, thereby improving data quality, and reduce the time required by mental health practitioners and clinicians for their administration and scoring [30]. In addition, the negative consequences of anxiety let emerge the need of short and easy-to-use instruments which could allow its detection at earlier stages, before that deleterious effects of chronic forms of anxiety occur [8].

A few short versions of GAS are currently available. The most recently developed was a German version of the GAS, named GAS-G-SF [31]. Validated both in the general population (*N* = 242) and medically ill sample (*N* = 156), this 9-item version showed good psychometric properties. However, its Somatic subscale exhibited lower reliability than the other two (Cognitive and Affective) subscales. Unlike the GAS-10, the GAS- G-SF was developed to be a multidimensional measure of geriatric anxiety. Specifically, three-factor model without any error covariance was provided an excellent fit by Gottschling et al. [31].

The 10-item GAS-10 version [32] was the most widespread short form of the GAS, developed and validated on a large US sample by using the Item Response Theory (IRT) approach. Excellent psychometric properties, including reliability, convergent validity, and unidimensionality, were reported by Mueller et al. [32]. More recently, Pifer and colleagues [33, 34] have developed an adapted 10-item version of the GAS (GAS-LTC), specifically tailored for long-term care settings. Starting from the already existing GAS-10, they slightly modified items according to specific cognitive needs of long-term care residents, as well as changed response format from an ordinal Likert-type scale to a dichotomous Yes/No response. Even though the initial validation was conducted in a small sample (*N* = 66), it showed good internal consistency and convergent validity.

Overall, both the unidimensional and multidimensional short forms developed so far appear to retain the amount of information captured by the longer version of the GAS, and confirmed their utility in detecting anxiety in older adults.

It is well know that unidimensionality is a desirable requirement for calculating and interpreting a total score of a clinical self-report instrument [35]. However, health outcomes measures are rarely strictly unidimensional [36]. This is due to the heterogeneous items that represent the complexity of health constructs [37, 38]. Although multidimensional instruments potentially reflected the heterogeneity of psychiatric disorders [39], unidimensional health measures were crucial in measuring the performance of health systems, evaluating outcomes in clinical trials and in everyday clinical practice [40]. To this purpose, flexible methodological approaches, as well as statistical models have been proposed to assess unidimensionality of health measures (eg. a bi-factor model) [41].

A brief, specific and unidimensional method of assessment of the severity of anxiety symptoms in older adults seems to be the answer to the main challenges posed by the measurement of anxiety in this population [13].

Currently, an Italian shortened unidimensional version of the GAS has not been developed yet, despite the current trend of growing percentage of elderly among the Italian general population and the increasing burden of mental disorders, including anxiety.

Therefore, the aim of the present study was to validate the Italian short-form of the GAS-10 in clinical and non-clinical samples. We conducted a series of separate analyses. First, we analysed the properties of the Italian version of the GAS-10 items by assessing within the IRT framework (*Structural analyses*). Next, in two independent samples of elderly people we investigated the concurrent validity of the Italian GAS-10 through a comparison with other validated instruments (*Concurrent validity analysis*). Finally, we tested the diagnostic performance of the GAS-10 with respect to Generalized Anxiety Disorder (GAD) (*Diagnostic performance analysis*).

## Materials and methods

### Samples

This study investigated the properties of GAS-10 administered to three independent samples: a community-dwelling older adults (samples 1 and 2) and a sample of geriatric outpatients with GAD diagnosis (sample 3). Samples characteristics were detailed in Table 1.

**Table 1:** Characteristics of the samples

***Samples***	Structural analysis	Concurrent and diagnostic performance analysis	
	Community (***N*** = 1200)	Community (***N*** = 229)	Clinical (***N*** = 35)
Female	645	*122*	*26*
Male	555	107	9
Age - M ± SD (Range)	65.21 ± 9.19 (50–92)	*65.01* ± *9.06* (53–91)	*74.89* ± *6.99* (62–89)
Marital status			
Single	90	*9*	1
Married	859	173	19
Divorced/separated	77	11	3
Cohabiting	10	*2*	*2*
Widowed	151	33	10
*missing*	13		
Education			
None	23	6	–
Primary school	268	42	11
Lower secondary school	301	54	11
Uppersecondary school	405	92	9
Bachelor/Master/Doctorate	155	28	4
*missing*	48		
	M ± SD	M ± SD	
ADL (Range 0–6)	5.88 ± 0.52	5.90 ± *0.47*	5.74 ± *0.51*
IADL (Range 0–8)	7.27 ± 1.18	7.35 ± *1.04*	6.94 ± *1.59*
MMSE	27.63 ± 2.21	27.37 ± *2.52*	28.69 ± *1.66*

*MMSE* Mini Mental State Examination, *SF12 – PCS* Physical Component Score; SF12, *MCS12* Mental Component Score, *ADL* Activities of Daily Living, *IADL* Instrumental Activities of Daily Living

**p < .001; *p < .01

#### Sample 1

The first sample consisted of 1200 Italian community-dwelling middle-aged and elderly adults (53.8% male, *N* = 645). Mean age of the total sample was 65.21 years (SD = 9.19; range = 50 to 92 years). Mean age for males and females was 64.94 ± 9.03 and 65.45 ± 9.32 (*t*(1,192) = .954; *p* = .341), respectively.

#### Sample 2

The second community sample included 229 community-dwelling elderly participants, of whom 46.72% were males. They were, on average, 65.01 (SD = 9.0) years.

In both the samples, subjects with cognitive and daily living skills impairment were excluded from the samples, by using the Mini Mental State Examination (MMSE); cut-off MMSE > 24 [42], the Activities of Daily Living (ADL) [43], and the Instrumental Activities of Daily Living (IADL) [44], respectively. Recruitment was through advertisements at several older adult associations. Study participants contributed voluntarily and anonymously; no honorarium was given for completing the assessments. All participants provided written informed consent, and filled out the questionnaires in-person using paper-and-pencil forms.

The methods inherent the assessment of the community sample were approved by the IRB of the Department of Psychological Sciences, Health and Territory, University of Chieti, Italy.

#### Sample 3

The clinical sample was composed of 35 elderly outpatients (74.28% were females; mean age of 74.89 ± 6.9 years) with Generalized Anxiety Disorder (GAD) according to DSM-TR criteria [40]. Cognitive functions and the ability to perform activities of daily living were preserved in the present clinical sample. They were recruited in the Psychiatric Clinic of the University Hospital of Udine, Italy, and diagnosed with the Structured Clinical Interview for Axis I DSM–IV disorders (SCID-I) [45]. Diagnoses were confirmed by the clinical consensus of two staff psychiatrists.

The assessment of the clinical sample was approved by the Ethics Committee of Friuli Venezia-Giulia Region, as part of a general study on the assessment of geriatric patients and their caregivers accessing the Psychiatric Clinic of the University Hospital of Udine, Italy (CEUR-2018-Sper-67-ASUIUD).

All participants provided their written informed consent to participate in this study.

### Instruments

#### Geriatric Anxiety Scale

The GAS long-form [14] Italian version [22, 27] is a 30-item self-report measure of anxiety symptoms among older adults. Participants were asked to indicate how often they have experienced each symptom during the immediately preceding week, including today. Response format uses a 4-point Likert scale ranging from 0 (not at all) to 3 (always), with higher scores indicating higher levels of anxiety. The GAS-long form covers three different domains of anxiety, which are common among older adults: somatic symptoms, cognitive symptoms, and affective symptoms. The GAS total score is based on the first 25 items. The additional 5 content items investigate areas of anxiety often reported to be of concern for older adults (eg. health and financial concerns). Cronbach’s alpha for the GAS-long form in the present study was good (.90).

A 10-item shortened version, the GAS-10, was developed, using Item Response Theory (IRT) approach, to reduce the burden of administration and scoring time, as well as to pose less burden on respondents [46]. The GAS-10 has strong evidence of reliability and validity for use with diverse samples of community-dwelling, medically ill, and treatment-seeking older adults [32, 33]. In the present study, Cronbach’s alpha was .84.

#### Geriatric Anxiety Inventory– short form (GAI-SF)

The GAI-SF [47] is a five-item self-report measure used to assess anxiety symptom severity among older adults. Respondents were asked to rate their agreement or disagreement with each item (yes/no response format). The measure was developed for mild cognitively impaired older adults and was derived from the 20- item GAI long form [47]. In the present study, Cronbach’s alpha was .70.

#### Geriatric Depressive Scale-15 (GDS-15)

Depressive symptoms were assessed by self-report with a visiting nurse assisted to read the questionnaire using the 15-item version of the GDS [48]. The GDS-15 is consisted of 15 items in a “yes/no” response format. Higher score indicates more severe depressive symptoms. In the current study, the internal consistency was high, with a Cronbach’s alpha coefficient of .78.

#### Short-Form 12-Item Health Survey (SF-12)

The Medical Outcomes Study Short-Form 12-Item Health Survey (SF-12) [49]; is a 12-item measure, assessed health related quality of life. The SF-12 is a shorter version of the SF-36 and has demonstrated similar psychometric properties [50]. It has also been validated to be used with older adults [51]. The measure includes two subscales, a Physical Component Summary score (PCS) and Mental Health Component (MCS) summary score, with higher scores indicating better physical and mental health, respectively.

### Statistical analysis

In the precision analysis on sample 1, construct unidimensionality was assessed through confirmatory factor analysis (CFA). The one-factor CFA model of the GAS-10 using the mean and variance-adjusted robust weighted least squares (WLSMV) estimator was evaluated using the Mplus 7 software [52]. The goodness of fit of the model was evaluated based on several fit statistics: (a) robust WLSMV chi square (χ2) statistic and its degrees of freedom; (b) Tucker Lewis Index (TLI); (c) comparative fit index (CFI); (d) root mean square error of approximation (RMSEA) and its 90% confidence interval (90% CI), and Weighted Root Mean Square Residual (WRMR). Due to the large sample size, interpretation of the robust WLSMV chi-square square as a measure of fit was eschewed. Values close to .06 for the RMSEA are indicative of a good fit, between .06 and .08 as moderate fit and values larger than .10 are indicative of a poor fit [53]. For the CFI and TLI, values of .95 or above indicate a good fit, whereas values between .90 and < .95 are taken as marginally acceptable fit [54].

According to the GAS-10 polytomous response format, and in line with the Mueller et al. [32] study, a Graded Response Model (GRM) [55]; was chosen. Although a number of different IRT models exists, the GRM logistic model assumes a single underlying trait (*θ*) and two item parameters: the difficulty parameter or threshold category (*b*) and the discrimination parameter (*a*). Under IRT, the difficulty of an item describes where the item functions along the trait, and it can be interpreted as a location index with regard to the trait being measured (the anxiety severity trait, in our case). Discrimination parameter, instead, describes how well an item can differentiate among adults with different levels of anxiety severity (*θ*). TIF and the related standard errors of measurement (SE) indicating the precision of the whole test [56] were also estimated to determine at what level of geriatric anxiety the GAS-10 provides the most information. The parameters were estimated by employing the Marginal Maximum Likelihood Estimation (MMLE) method with the Expectation–Maximization algorithm (EM) [57]. All IRT analyses were conducted using the *mirt* package in R [58].

Local independence (LI) (the assumption that the only influence on an individual’s item response is that of the latent trait variable being measured and that no other variable is influencing individual item responses) was also assessed. A violation of the LI assumption can distort estimated item parameters, item standard errors, and model-fit statistics [59, 60]. To assess the tenability of LI, the standardized LD-χ^2^ statistic, a local dependence matrix based on the χ^2^ statistics [61], for each item was examined. Standardized LD residuals were expressed in the form of signed Cramers V coefficients. A value of |10| or greater is considered large and reflecting LI issues [52]. LD- χ^2^ statistics were calculated using the residuals function in *mirt*.

Logistic regression was used to investigate DIF based on the latent anxiety estimates derived from IRT using the *lordif* package in R [57]. Effect size estimation for DIF was computed using the expected test score standardized difference (ETSSD) index [62], implemented in *mirt* package with the empirical_ES function. The ETSDD can be interpreted using the guidelines on effect size given by Cohen [62, 63].

In order to test the adequacy of the model we computed the C_2_ fit statistic [64], available using the M2 function in the *mirt* package. The C_2_ is a limited-information goodness of fit test statistic for ordinal IRT models, and is mainly useful when the number of items is small and the number of categories is large. The goodness of fit of the GRM model was evaluated based on several fit statistics (such as CFI, TLI, RMSEA, and SRMR) that are strictly related to those in structural equation modeling (SEM and CFA) (*Model Diagnostic*). Information curves, ICCs, threshold parameters, and discrimination parameters were analyzed to examine the item properties of the measure and identify which items were more or less useful in reliably measuring trait levels of anxiety. Discrimination parameter (α) values theoretically range from −∞ to ∞, but from .50 to 3.0 represents a reasonably good range. Difficulty parameter (*b*) values ranging from − 3 to + 3 are in the typical range; and − 2.8 to + 2.8 are in the usual range [65].

The concurrent validity and diagnostic accuracy of the GAS-10 were conducted using two independent samples of elderly adults, a sample of community-dwelling healthy adults and a outpatient sample of adults with GAD (sample 2 and 3). To investigate concurrent validity of test score interpretations, Pearson correlations were calculated between scores on the GAS-10 and scores on the GAS-long form, the GAI-SF, the GDS-15, and the SF-12. In order to provide an accurate estimation of the associations, the GRM based latent trait scores were estimated for the GAS-10 concurrent validity analysis. We also compared the GAS-10 and GAS-long form pairs of correlation coefficients in the analysis of concurrent and discriminant validity following Meng et al. [66]. This procedure involves performing a Fisher *Z* transformation on the correlation coefficients so that they can be compared via a t-test.

The diagnostic performance of the 10-item GAS was assessed using the Area Under (AUC) the Receiver Operating Characteristic curve (ROC). The Youden (J) method was employed in order to detect the cut-off score of the GAS-10. ROC curve analysis was done using the MedCalc software package [67]. Optimal values of AUC ranged from 0 “weak performance” to “1” perfect performance”, with a values of 0.7 to 0.8 are considered “fair,” 0.8 to 0.9 “good,” and ≥ 0.9 are considered “excellent” [68]. Belonging to the non-clinical and clinical sample (dichotomized as 0/1), and the GAI-SF cut-off score (> 2) [69] were employed to classify participants with low and high anxiety symptoms. We also computed a series of pairwise comparison of ROC curves to test whether the GAS long and short forms differed in performance across diagnoses.

## Results

### Structural analyses

As a preliminary step, unidimensionality of the geriatric anxiety construct was assessed on sample 1 with a CFA using the WLSMV. Results showed that the 10 GAS items measured one dimension, WLSMV χ^2^ (35; *p* < .001) = 329.683, CFI = .95, TLI = .95, and RMSEA = .08 CI = [.076, .092], WRMR = 1.63. All factor loadings were significant (*p* < .001), ranging from .47 to .82. Having verified the assumptions that a single continuous construct accounted for the covariation between item responses, unidimensional GRM-IRT analyses were performed. The GRM was applied in order to estimate the item discrimination (*a*) and difficulty (*b*) parameters, in line with the Mueller et al. study [32]. The range of item discrimination (α) was between 0.941 ± .09 and 2.618 ± .22 logit (Table 2). The variation in discrimination parameter suggested that a GRM estimating unique slope parameters for each items was good for our data. Thus, the items can adequately distinguish among individuals with different levels anxiety. In line with the discrimination parameters found in Mueller et al. [32], items from somatic subscales (eg. item #4, *a* = .940 ± .09) tended to discriminate less than affective and cognitive subdomains. The three *b* difficulty parameters indicated noticeable moderate to large increase in the level of the latent trait at each subsequent response category; that is, all items showed higher values in the level of the latent trait (anxiety) at each subsequent response category. For all items, the *b*_1_, *b*_2_, and *b*_3_ values were quite evenly spaced, with *b*_1_ the close to the mean trait (fixed at M = 0.00, SD = 1.00, by default), *b*_2_ and *b*_*3*_ above it. Specifically, the *b*_2_ values were close to the 2SD above the mean trait level, the *b*_3_ values were close to the 3SD above the mean trait level (Table 2). In sum, the GAS-10 items perform well in measuring a large spectrum of the underlying construct.

**Table 2:** Item discrimination (*a*) and difficulty parameter (*b*) estimates along with standard errors (±*SE*) for each item of the GAS-10

GAS-10 *Item*	***a ± SE***		***b***_**1**_ ***± SE***		***b***_**2**_ ***± SE***		***b***_**3**_ ***± SE***	
Item #4	0.941	±.09	1.538	±.14	3.271	±.30	4.448	±.43
Item #1	1.446	±.10	−0.619	±.06	2.267	±.14	3.589	±.25
Item #8	1.496	±.17	−0.088	±.04	1.658	±.08	2.691	±.13
Item #7	1.527	±.16	−1.246	±.06	1.092	±.12	2.290	±.24
Item #2	1.528	±.12	0.836	±.07	2.660	±.17	3.843	±.29
Item #5	1.750	±.10	0.321	±.05	1.905	±.10	2.851	±.16
Item #10	2163	±.16	0.992	±.06	2.157	±.11	2.786	±.16
Item #3	2.193	±.10	0.869	±.08	2.309	±.07	3.439	±.13
Item #6	2.508	±.12	−0.012	±.05	1.592	±.10	2.512	±.17
Item #9	2.618	±.22	1.230	±.06	2.401	±.13	3.068	±.20

The goodness of fit for GRM model indicated a good-to-acceptable model fit for the total sample, C_2_ (35; *p* < .001) = 247.1031, CFI = .97, TLI = .96, and RMSEA = .07 CI = [.062, .079], SRMSR = .04. Using guidelines developed in the context of linear factor analysis and structural equation modeling for continuous data, the SRMSR(≤.05), TLI and CFI(≥.95) indexes were above the threshold of the acceptability, and the RMSEA within the acceptability range (.05 < RMSEA>.08) [54].

**Fig. 1:**
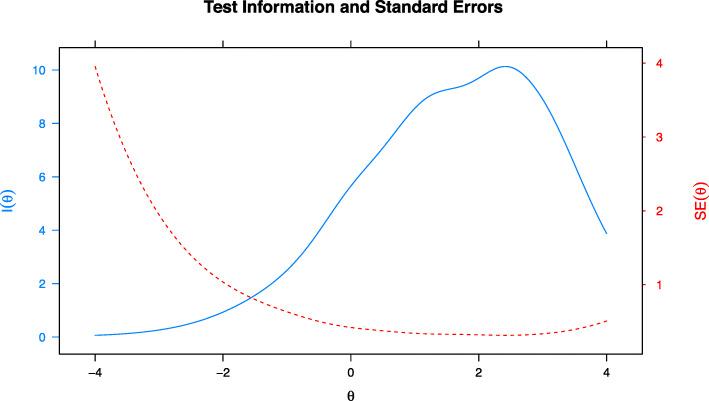
Total information function (solid line) and standard error of estimate (dashed line) for the GAS-10

Moreover, in order to identify the area of trait that is accurately assessed by the scale, the TIF was analyzed. Figure 1 displays the TIF for the 10-item GAS scale. The TIF shows the GAS-10 provided a relative uniform information (information ≅ 8.9) for the range 0.5 to 3 logits, which has an associated empirical reliability of about .81 and standard error of estimate of about ≅.33.

Then, the GAS-10 was tested evaluating the presence of LI, to assess an excess of covariation among item responses that is not accounted for by a unidimensional IRT model. Results showed that a single factor model adequately represented the structure of the GAS-10 given that none of the LD statistics were greater than |.3|.

DIF was examined for each item across gender (males vs females) and age (under70 vs over70) groups, performing an ordinal (common odds-ratio) logistic regression DIF analysis using IRT theta (θ) estimates as the conditioning variable. Results showed no DIF for gender. Concerning the age groups, three items displayed DIF (#1: “*I was irritable*”, #3: “*I felt like I was in a daze*”, #6: “*I felt restless, keyed up, or on edge*”). However, an examination of the McFadden’s pseudo R^2^ criterion showed that the magnitude of DIF for these items was negligible (close to .02) [64]. At the overall test level there is minimal difference in the total expected score at any anxiety level for under or over 70 aged participants, with a small effect size (ETSSD = .23).

### Concurrent validity analysis

Differences in anxiety, and depression variables, as well as in health dimensions, were found among non-clinical (sample 2) and clinical samples (sample 3). Means and standard deviations, group *t*-tests were provided for each independent variable in Table 3. Pairwise deletion techniques were applied to handling missing data. As expected, clinical adults exhibited significantly higher levels of anxiety, as measured by GAS-long form and the GAS-10 [*t*(262) = − 4.80/− 4.28, *p* < .001]; GAI-SF [*t*(262) = − 6.01, *p* < .001], and depression [GDS-15; t(262) = − 6.56, *p* < .001]. On the other hand, non-clinical sample showed high level of physical [PCS; *t*(262) = 2.69, *p* < .01] and mental [MCS; t(262) = − 6.01, *p* < .001] health compared to the clinical sample. Both the GAS-long form and the GAS-10 scales were highly inter-correlated (*r* = .806, *p* < .001). Additionally, GAS-10 was moderately correlated with the GAI-SF (*r* = .570, *p* < .001), the GDS-15 (*r* = .540, *p* < .001) scales, and the Mental Component Score of the SF-12 scale (*r* = −.418, *p* < .001). No significant correlation was found between the GAS-10 and the Physical Component Score of the SF-12. Subsequently, correlation coefficients between the GAS-long form and GAS-10 with GAI-SF, and GDS-15 were statistically compared (Table 3) [66]. Comparisons revealed that the GAS-long form and the GAS-10 correlated equally with GAI-SF scores [*t*(261) = 1.49, Z = 1.48], as well as with the GDS-15[*t*(261) = .57, Z = .57].

**Table 3:** Descriptive, differences and correlations among GAS-long form, GAS-10, GAI-SF, GDS, and SF-12

	Community (***N*** = 229)	Clinical (***N*** = 35)		***Correlations***				
	M ± SD		***t***-test	***2.***	***3.***	***4.***	***5.***	***6.***
1. GAS-Long form	15.18 ± *9.26*	23.60 ± *12.01*	−4,80**	.806^**^	.558^**^	.523^**^	−.036	−.360^**^
2. GAS-10	5.04 ± *4.07*	8.46 ± *6.07*	−4.28**^,a^		.540^**^	.570^**^	−.056	−.418^**^
3. GDS-15	3.59 ± *2.95*	7.17 ± *3.38*	−6.56**			.496^**^	.070	−.356^**^
4. GAI-SF	2.06 ± *1.56*	3.71 ± *1.20*	−6.01**				.004	−.440^**^
5. SF-12 PCS	41.12 ± *6.33*	37.94 ± *6.37*	2.69*					−.345^**^
6. SF-12 MCS	41.53 ± *6.52*	36.24 ± *5.83*	4.40**					

*GAS* Geriatric Anxiety Scale, *GDS* Geriatric Depression Scale, *SF12 – PCS* Physical Component Score, *SF12 - MCS12* Mental Component Score

**p < .001; *p < .01

^a^t-test based on latent scores

### Diagnostic performance analysis

A first ROC curve analysis was performed to compare the elderly adults with high versus the low anxiety symptoms risk group based on the clinical/non-clinical sample (samples 2 and 3). The results indicated that the 10-item GAS scale was able to discriminate between the two groups (Table 4). The AUC for the GAS-10 total score was .702 (95%CI = .643–.757), suggesting fair discrimination between the groups. The *J* index of .35 for the GAS-10 total score was observed at a score of 6 points, corresponding to sensitivity of 62.86% and specificity of 72.93%. The positive and negative predictive powers were 26.2 and 92.8%, respectively.

**Table 4:** Area Under the Curve (AUC) of the Receiver Operating Characteristic Curve (ROC) Analyses for the different versions of the GAS, and Comparison of independent ROC curves

Criteria	AUC	SE	(95%CI)	Sensitivity/	Cut-off	PPV/NPV
				Specificity		
Clinical vs NonClinical						
GAS-long form	.729	.045	.672/.782	91.43/28.82	> 9^a^	16.4/95.7
				88.57/51.53	> 13	21.8/96.7
GAS-10	.702	.046	.643/.757	62.86/72.93	> 6	26.2/92.8
*ROC curve comparison*	∆AUC	SE	(95%CI)	z	*p*	
GAS-long form vs GAS-10	.026	.035	−.041/.095	.780	.435	
GAI-SF > 2						
GAS-long form	.797	.028	.743/.844	91.82/38.96	> 9^a^	51.8/87.0
				71.82/75.97	> 15	68.1/79.1
GAS-10	.745	.030	.688/.797	53.64/83.77	> 6	70.2/71.7
*ROC curve comparison*	∆AUC	SE	(95%CI)	z	*p*	
GAS-long form vs GAS-10	.052	.0233	.006/.097	2.226	*.026*	

^a^ cut-off identified by Gould et al., 2014 [70]

Similarly, a second ROC curve was performed to compare the older adults with low versus high anxiety symptoms as assessed by the GAI-SF cut-off (> 2) [69]. The results indicated that the GAS-10 scale was able to discriminate the two groups with an AUC of .745 (95%CI of .688–.797). The *J* index of .37 for the GAS-10 total score was observed at a score of 6 point, corresponding to sensitivity of 53.64% and specificity of 83.77%. The positive and negative predictive powers were 70.2 and 71.7%, respectively. Thus, the GAS-10 cut-off score remained stable at 6 points despite the different classification criteria applied. This cut off point provided a balanced ratio between sensitivity and specificity, and correctly diagnosed elderly adults at high risk of anxiety symptoms compared to those with a low anxiety symptoms risk.

Far from what has been observed with the GAS-10, diagnostic accuracy of the GAS-long form were achieved with different cut-off scores in our sample (> 13 and > 15), displaying high sensitivity values. The high sensitivity of the GAS-long form remained high even if additional cut-off score in literature was applied (> 9) [70]. When we compared the predictive validity of the total scores of the GAS-long form and the GAS-10 model, the AUCs were quite identical (ΔAUC = .026), and the differences between them were not found to be significant for those were diagnosed with GAD or for those were healthy. Subsequently, when we considered the GAI-SF as a diagnostic criterion, the predictive validity of the total scores of the GAS-long form and the GAS-10 model differed significantly at value of *p* < .05 (ΔAUC = .052). Thus, results of the pairwise comparison revealed that GAS-10 did not differ in diagnostic accuracy from its long form, when classification criterion was based on sample characteristics (clinical vs non-clinical). Slightly differences were displayed between them, if GAI-SF measure was applied to classify elderly adults with high from low anxiety symptoms.

## Discussion

Given the overlapping of symptoms of mental disorders (eg. depression and anxiety) in later life and the comorbidity with physical health problems, it is important to design unidimensional anxiety measures that have been specifically targeted for older adults that account for and measure this unique expression [13, 23]. This is also supported by the controversial findings derived from empirical studies on the composite ‘general distress score’, which combines depression and anxiety symptoms in a unidimensional domain [71]. This study aims to filling this gap, adding evidence of the adequacy of the GAS-10 for the assessment of geriatric anxiety through IRT-based evidence.

The IRT analysis provided a clear framework of the good performance of the GAS-10 items in measuring the geriatric anxiety construct. In accordance with Mueller et al. [32] study, our study showed that the GRM model accurately explained the pattern of responses obtained by the GAS-10 scale. In deep, the item parameters showed that all the GAS-10 items were able to distinguish adequately among older adults with different levels of the trait being measured, and adequately covered the spectrum of the latent trait. Concerning the item difficult values, our findings showed that the GAS-10 items had a medium to high level of difficulty, indicating a decreasing of the probability to endorse the response option “*all the time*” to self-statements that quantify the frequency that anxiety symptoms. Nonetheless, the discrimination parameter values in the present study ranged from “low” (item #4) to “moderately high” (item #9), suggesting that the GAS-10 items can discriminate older adults with different anxiety symptoms severity levels. Compared to the cognitive and affective items, the somatic items were found less informative. These findings had already been reported in previous studies employing both the GAS-10 [32] and other anxiety measures, such as the State-Trait Inventory for the Cognitive and Somatic Anxiety (STICSA) [21]. It is still open the debate among researchers about the inclusion of physiological symptoms items in anxiety measures, mainly in the elderly, since these could undermine the uniformity of the anxiety construct. Indeed, the content of somatic items in anxiety measures have been criticized due to the possible overlap with the depression symptomatology [30]. In fact, somatic symptoms (eg. fatigue, aches and pains) have been found to be a prominent part of the clinical presentation in depressed older adults [72]. In addition, the co-occurrence of physical health problems in older adults makes differentiating between somatic symptoms of anxiety, and depression difficult [73].

The GAS-10 adequately measured geriatric anxiety ranging from medium to high levels, whereas it was less precise for the lowest levels of the trait. To date, the highest informative point of the GAS-10, or the peak, was observed at 2.5 standard deviation above the mean level of anxiety, supporting its use as a clinical meaningful measure to assess elderly who experienced high levels of mental distress.

Far from previous study the DIF analysis for each item across gender confirmed the invariance of the GAS-10, providing evidence of its ability to scale males and females onto a common metric. Commonly, females tend to score highly in the GAS total score than males, since they are more comfortable in to express mental health symptoms, as well as showed more risk factors [74, 75]. Potentially, this unbiased version of the GAS-10 could allow an easy and efficient assessment of anxiety among elderly, without the use of differentiated norms by gender. On the contrary, DIF has been found across age, despite its impact was negligible and close to the 2% of the items true score. Adults aged over 70 years scored higher on three items, two of these were drawn from the affective and one from the cognitive domain of the GAS-10. DIF of affective anxiety items in older adults, as well as in the cognitive item measuring daze/confusion, were likely to affect by the higher prevalence of depressed mood symptoms [76].

Additionally, the GAS-10 has been administered to test the validity and the diagnostic accuracy of the scale in two samples of healthy older adults and outpatients with GAD diagnosis. Similar to the GAS-long form, independent *t*-test analysis revealed that the GAS-10 was able to capture differences in anxiety symptoms between the two samples. As expected, outpatients with GAD diagnosis displayed statistically higher mean scores of the GAS-10, compared to the healthy sample. Results from correlation analysis, also, provided good support for the validity of the GAS-10. The brief and the long version of the GAS were found highly interrelated, and pairwise comparison analysis corroborated the high degree of overlap between them in correlating with concurrent validity measures.

In line with Gottschling et al. [31], both the GAS-10 and its long form were found to correlate on average with depression (as measured by the GDS), thus confirming the co-occurrence of anxiety and depression symptomatology among elderly. In addition, this finding was not surprising, since the GDS has been criticized for its lack of unidimensionality, and construct validity [13].

The diagnostic performance of the GAS-10 was assessed by means of ROC analysis to detect elderly with clinically significant GAD symptoms, as well as with significant anxiety symptoms, as measured by the GAI-SF cut-off scores. Differences in diagnostic accuracy between the long form version of the GAS and the GAS-10 was not found significant, when clinical/non-clinical classification variable has been taken into account. An optimal cut-off value score > 6 for the GAS-10 was selected to maximize the sum of sensitivity and specificity. Therefore all the patients with a total score of 6 in the GAS-10 should be referred for further risk assessment and management. When GAI-SF cut-off has been implemented as classification variable in the ROC analysis, the GAS-10 cut-off remained unchanged, preserving a discrete specificity. Our findings are in line with the previous studies, in which a cut-off score of > 9 optimized the balance of sensitivity and specificity (.60 and .75), for the GAS –long form [70]. Likewise, diagnostic accuracy of the GAS- G-SF in clinical settings was low, showing a sensitivity of 70% and a specificity of 30% (cut-off > 5) [31].

Nevertheless, the Italian version of the GAS-10 could be considered a stable measure of geriatric anxiety, with a sufficient discriminant validity in categorizing outpatients diagnosed with GAD, and adults with clinical significant anxiety symptoms (as classified by the cut score of the GAI-SF). Although its use as a screening test might be limited, the identification of a clinical cut-off score of the GAS-10 could help clinicians and researchers to identify older adults with anxiety disorders in resource-constrained settings, where time constrain and fatigue issues affect clinical assessment validity. In this direction, future studies with larger clinical samples are needed to support the clinical utility of the GAS-10.

Some limitations of this study could be addressed in future research. First, participants in the present study were community-dwelling participants and outpatients’ elderly with GAD diagnosis. There is also evidence that medically ill samples of older adults experienced severe levels of anxious distress [23, 77], and were most likely to overburden healthcare services [46, 78, 79]. Thus, further studies could address GAS-10 diagnostic accuracy in samples of older adults with GAD diagnosis in comorbidity with medically ill patients. A second limitation was the high prevalence of females in our clinical sample. This datum, however, was similar to real life, since being female gender was found one of the principal factor associated with the pure GAD [80, 81]. Another limitation of this study was its cross-sectional design which limits causal inferences, and make difficult to understand if the GAS-10 items are a stable anxiety symptoms measure. Further longitudinal studies are required in this direction.

## Conclusion

Our study provided support for the psychometric functioning of the GAS-10, and its diagnostic performance in discriminate accurately older adults with GAD from elderly adults without significant clinically relevant symptoms of anxiety. In this sense, the present study improved upon previous studies by using a rigorous psychometric technique to validate the short measurement, the Item Response Theory (IRT) [82].

Indeed, differently from the Classical Test Theory (CTT), the IRT considers the characteristics of both the test and the sample, discarding items which are not endorsed by respondents and thus do not contribute significantly to the latent-attribute scores [37, 56, 83, 84].

Through the use of IRT, this study provided additional information about the psychometric functioning of the GAS-10 and some cues for improving its clinical utility. Our results could increase the confidence in the assessment accuracy of the GAS-10 and give support to the huge amount of studies that employed it in the field of geriatric anxiety.

## Data Availability

The datasets used during the current study are available from the corresponding author on reasonable request.
